# Raynaud's Disease Affecting Tongue as Well as Extremities

**Published:** 1907-10-12

**Authors:** 


### AN UNUSUAL CASE OF RAYNAUD'S DISEASE.

### The Tongue as well as Extremities Affected.

The three degrees of Raynaud's disease—local syncope, local asphyxia, and local gangrene—are well enough known, and cases exhibiting the first and second degrees of the trouble in the fingers and toes are not very uncommon; fortunately the third stage, which is a sad condition, is very much rarer. The following is an example of it, together with Raynaud's disease of the tongue at the same time.

The patient is a woman now aged 44; there is nothing notable about her family history, and, except for the ordinary ailments of childhood, she was perfectly well except for occasional neuralgia in various parts of her head, until she was twenty-eight. She then had her first and only confinement, which was rather long and troublesome. Three months afterwards, when she had become quite strong again, she noticed during March that the little finger of her right hand became very cold and white, and painful to pressure.

Dipping the hand into hot water, or keeping it in a warm glove, cured the local syncope for the time being, but immediately the hand was allowed to get at all cold the little finger at once became blanched and painful again. Six weeks later this troublesome finger, instead of merely going blanched, began to turn livid, and presently purple. The local asphyxia lasted a month, the bluish-purple colour deepening, until one day a breach of surface occurred, followed by a discharge of dark and fœtid pus. The pain in the affected finger was intense. The suppuration lasted about six months, and then the place healed over, and the digit recovered a pinkish, almost normal colour. Very soon after this precisely similar changes occurred in the ring finger of the same hand, lasting about the same length of time, and occurring in the same order. The middle finger, and then the index finger, followed suit.

As time went on, the left hand suffered from the affection too, the order in which the left digits succumbed being: index, middle finger, ring finger, and little finger. Presently the local asphyxia, instead of being recurrent, became permanent, and the nails became corrugated and rough from irregular formation. When the patient was 39 years old the thumbs, which up to then had escaped altogether, became involved too. When she was 41 two of the fingers suppurated simultaneously.

All this time the feet, though very sensitive to cold, had never been affected by local syncope or local asphyxia; a year ago, however, that is to say at 43 years of age, the toes became swollen and painful, and there were cramps in the legs; the changes in colour in the toes were not noticed much by the patient herself, because she kept them well wrapped up; but on attention being directed to them they were found to suffer from paroxysmal syncope and asphyxia precisely similar to those of the fingers, and on one occasion the little toe of the right foot suppurated spontaneously for some days.

The nose and ears were not affected at all.

At present the patient is thin and pigmented, the right thumb is swollen, red, and painful, and almost the whole of its terminal phalanx is black and gangrenous, the necrosis involving the bone as well as the soft parts. The index has lost much of the tissue over its second phalanx, and its third phalanx is represented by little more than a deformed nail. The middle finger is semi-ankylosed, and its terminal phalanx has disappeared except for a small and deformed nail. The ring finger is gone altogether. The little finger is twisted and altogether deformed. The left hand digits are all atrophic, cyanosed, and painful, and each has lost almost the whole of its terminal phalanx; at the ends of the thumb and index finger there is a tiny corrugated nail; the ring finger is the only one that has retained anything like its normal size, and even here there is extreme deformity by acute flexion at the second interphalangeal joint. The feet present more acute lesions of a very similar nature.

The heart, lungs, abdominal organs, and urine are all natural. The patient has a very fair appetite, and the complexion is good. As regards the general nervous system, the tendon reflexes are all exaggerated, though there is no ankle clonus, and the plantar reflex is flexor; the pupils react normally; the sphincters are natural. When reading, the patient often complains of a mist coming before her eyes, so that she has to lay down her book for twenty minutes or half an hour before she can go on with it, but the optic discs look perfectly healthy, and vision otherwise seems natural.

Sensation seems to be natural, and nowhere is there any loss of sensibility to heat and cold, or to pain, so that syringomyelia cannot be diagnosed. There has been no alcoholism, nor is there any evidence of lead poisoning, nor of ergotism; so that Raynaud's disease seems clearly to be the diagnosis.

As has been said, the trouble has now begun to affect her tongue as well as her hands and feet; and this makes her much more miserable. Suddenly, without any obvious cause, and apart from any apparent emotional disturbance, she feels her tongue go cold; she says it feels as though she had an ice in her mouth. The phenomenon lasts a quarter of an hour, during which time she moves her tongue about, as though chafing it warm again. She feels it tingling, and any strong pressure on it is acutely painful. The end of the attack is ushered in by violent and intensely painful throbbings, which begin at the tip, and appear to be precisely similar to those experienced in hands that are recovering from exposure to extreme cold. The attack is often preceded by pain arising in the supra-hyoid region, and thence spreading from behind forward into the lingual region.

When the tongue is examined during one of these crises it is a deep violet colour, and the livid tint lasts some time after the actual attack has passed off. The lingual seizures occur once or twice a week; fortunately they have not hitherto led to any suppuration or gangrene of the tongue, but one fears that this result is very liable to follow some day. Treatment for the condition can do little more than mitigate the patient's sufferings.

